# The century-old picture of a nerve spike is wrong: filaments fire, before membrane

**DOI:** 10.1080/19420889.2022.2071101

**Published:** 2022-05-10

**Authors:** Subrata Ghosh, Pushpendra Singh, Jhimli Manna, Komal Saxena, Pathik Sahoo, Soami Daya Krishnanda, Kanad Ray, Jonathan P. Hill, Anirban Bandyopadhyay

**Affiliations:** a Chemical Science and Technology Division, CSIR-North East Institute of Science and Technology, NEIST, Jorhat, India; b Academy of Scientific and Innovative Research (ACSIR), Ghaziabad, India; c International Center for Materials and Nanoarchitectronics (WPI-MANA), Research Center for Advanced Measurement and Characterization (RCAMC), Tsukuba, Japan; d Advanced Technology Development Center, Indian Institute of Technology, Kharagpur, India; e Microwave Physics Laboratory; Department of Physics and Computer Science, Dayalbagh Educational Institute, Dayalbagh, India; f Amity School of Applied Science, Amity University Rajasthan, Jaipur, India

**Keywords:** Nerve spike, Ion channel, neuron, neurophysiology

## Abstract

In 1907, Lapicque proposed that an electric field passes through the neuronal membrane and transmits a signal. Subsequently, a “snake curve” or spike was used to depict the means by which a linear flat current undergoes a sudden Gaussian or Laplacian peak. This concept has been the accepted scenario for more than 115 years even appearing in textbooks on the subject. It was not noted that the membrane spike should have a cylindrical shape. A nerve spike having a dot shape on membrane surface cannot propagate through a cylindrical surface since it would dissipate instantaneously. A nerve spike should have the appearance of a ring, encompassing the diameter of a cylindrical axon or dendron. However, this subtle change has remarkable implications. Maintaining a circular form of an electric field is not easy, especially at the surface of an organic object. Here, we suggest that neuroscience could redefine itself if we accept that a nerve spike is not a localized 3D Gaussian or Laplacian wave packet, rather it is a 3D ring encompassing the diameter of a neural branch.

Some insightful experiments have occasionally been made on the subject of this review, but those studies have had almost no impact on mainstream neuroscience. In the 1920s (Katz, E. [1]), it was shown that neurons communicate and fire even if transmission of ions between two neighboring neurons is blocked indicating that there is a nonphysical communication between neurons. However, this observation has been largely ignored in the neuroscience field, and the opinion that physical contact between neurons is necessary for communication prevailed. In the 1960s, in the experiments of Hodgkin et al. where neuron bursts could be generated even with filaments at the interior of neurons dissolved into the cell fluid [3,4], they did not take into account one important question. Could the time gap between spikes without filaments be regulated? In cognitive processes of the brain, subthreshold communication that modulates the time gap between spikes holds the key to information processing [14][6]. The membrane does not need filaments to fire, but a blunt firing is not useful for cognition. The membrane’s ability to modulate time has thus far been assigned only to the density of ion channels. Such partial evidence was debated because neurons would fail to process a new pattern of spike time gaps before adjusting density. If a neuron waits to edit the time gap between two consecutive spikes until the density of ion channels modifies and fits itself with the requirement of modified time gaps, which are a few milliseconds (~20 minutes are required for ion-channel density adjustment [25]), the cognitive response would become non-functional. Thus far, many discrepancies were noted. However, no efforts were made to resolve these issues. In the 1990s, there were many reports that electromagnetic bursts or electric field imbalance in the environment cause firing [7]. However, those reports were not considered in work on modeling of neurons. This is not surprising because improvements to the Hodgkin and Huxley model made in the 1990s were ignored simply because it was too computationally intensive to automate neural networks according to the new more complex equations and, even when greater computing powers became available, these remained ignored. We also note here the final discovery of the grid-like network of actin and beta-spectrin just below the neuron membrane [26], which is directly connected to the membrane. This prompts the question: why is it present bridging the membrane and the filamentary bundles in a neuron?

The list is endless, but the supreme concern is probably the simplest question ever asked in neuroscience. What does a nerve spike look like reality? The answer is out there. It is a 2D ring shaped electric field perturbation, since the ring has a width, we could also state that a nerve spike is a 3D structure of electric field. In Figure 1a, we have compared the shape of a nerve spike, perception vs. reality. The difference is not so simple. Majority of the ion channels in that circular strip area requires to be activated simultaneously. In this circular area, polarization and depolarization for all ion channels should happen together. That is easy to presume but it is difficult to explain the mechanism. How could membrane control all ion channels in a ring shape and propagate the ring linearly along the membrane? Further to this problem, whenever a new phenomenon is observed in neuroscience, a single parameter (the density of ion channels) is invoked to account for it, and other explanations are not sought. For example, in the 1960s, branch selection was introduced as a mechanism by which to understand brain cognition, since it mapped synchronized, phase coupled or on-off correlated synaptic junctions [10]. While certain neural branches are selected when a nerve spike transports, some are also rejected. Initially, this was attributed to variations in the diameters of the branches then, finally, to the density of ion-channels. Synaptic junctions of a neuron do not all acquire identical states when a neuron fires. A burst is neither random nor homogeneously transported through all junctions. As the neuron bursts, for the transmission of a nerve spike, some of the synaptic junctions switch on and off, and both groups of junctions synchronize in a hierarchical group. For a specific time range, a group of junctions becomes active, are then silent for a period, and then return to an active state. Again, the complex selection and deselection of groups of junctions have been attributed to the density of ion channels. It is difficult to list the many phenomena attributed to the characteristics of ion channels.

**Figure 1.**
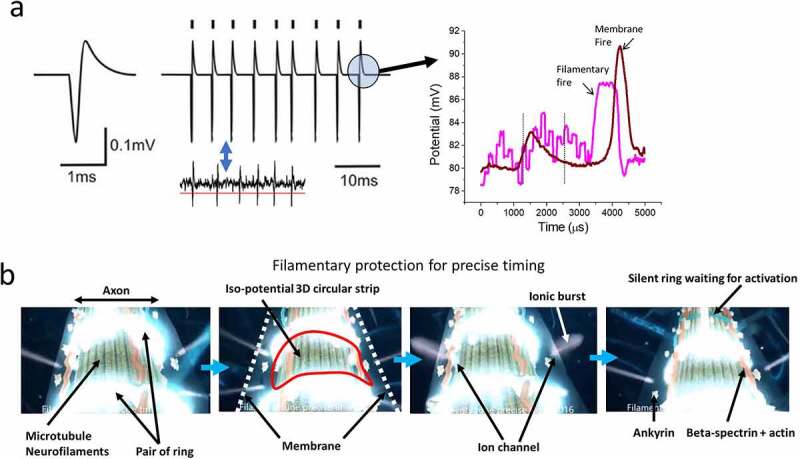
(a) Comparison of a classic textbook representation of a nerve spike and (b) most probable structure in reality as per state-of-the-art studies.

In the case pertinent here, the circular shape of a nerve spike has also been attributed to ion channel density. A possible explanation would be that a ring-shaped area should have an equal density of Na, K, and Ca ion channels, although that has not been observed experimentally. This demand contradicts the ion channel density requirement for branch selection problem. However, there is an additional concern. In the existing CMOS-based computer chips, the fastest clocks group together to create longer and slower clocks. There is no known scientific mechanism following which one could control a millisecond clock using resonators or elements that vibrates in the millisecond time domain. To precisely control ion channels, a microsecond or faster clock is required, which does not introduce millisecond errors. The neuroscience literature contains many reports addressing events operating in the millisecond, nanosecond, picosecond or femtosecond domains, and reports on the corresponding ac frequencies are also abundant. Notably, the microsecond time domain is absent from this list. For this reason, it was essential to bridge this missing frequency gap in MHz. While assigning a backup clock for constructing the streams of milliseconds pulses governing the key cognition signals; a suitable MHz component was not found for operation in the microsecond domain.

Therefore, around 2008, investigations on microtubules revealed that they are capable of reversibly writing and storing information [18–20]. If microseconds pulses are sent through a single microtubule, the transmitted electric field across a single microtubule is amplified by a factor of 10^4^ depending on the time width of pulses and the time gap between the pulses. It is difficult to conceptualize that time modulation could be tested by sweeping the frequencies of ac signals we shined on microtubules using an antenna. Frequencies that cause bursts in electric fields are resonance frequencies. Quantum tunneling images reveal the appearances of proteins or filaments when they vibrate under resonance ([20], Movie 1). All the 12 major frequencies form triplet of triplet groups where the microtubule bursts pulses for microseconds; peaks nearby in the transmission spectrum form groups. Importantly, the grouping of resonance frequencies is scale-free [16]. This means that individual proteins, filaments composed of proteins, and whole neuron cell groups spontaneously resonate at similar groups of frequencies. Triplet of triplet groups are always found irrespective of the frequency domains [9]. For example, for the frequency bands 1–40 Hz, 1 kHz-40 kHz, 1 MHz-40 MHz, 1 GHz-40-GHz, three wide resonance peaks in each frequency band can be observed which, if observed more closely, each contain three further peaks.

Moreover, each resonance frequency is associated with a phase shift. While passing through a microtubule nanowire, the ac signal shifts its phase by nπ/4, (n = 0,1,2 …). The sum of phase shifts for a set of resonance frequencies forming a group is 360°, so completing a phase cycle is not incidental. Microsecond long bursts integrate time lapse to form a new slower clock, which is required to support the millisecond pulses. Synthesizing clocks, integrating these clocks, and building 3D clock assemblies are fundamental properties of all major filaments, their associated proteins, and neurons [5,9,15]. The time crystal concept was introduced in the 1960s by A. Winfree to explain the intelligence of viruses. A periodic burst that is generated purely from internal mechanisms and in spite of external perturbations, the internal clock is regenerated. The concept was forgotten but was revived in 2013 by Frank Wilczeck. However, both proposals suggested that internal clock frequency where time symmetry breaks are integral to an external perturbation. We generalized the concept to clocks having different diameters or periodicities, which operate coherently, and the term “polyatomic time crystal” has been coined to describe this [17]. However, 3D clock assembly or time crystal [15] suggests that distinct structures inside neurons and membranes are phase correlated, although physical measurements on this subject are scarce. The concern now is that, if microtubule-like filaments control the precise time width of ion spikes, are microsecond long bursts of filaments correlated with the milliseconds’ bursts of neurons?

The correlations between filamentary electromagnetic ac signals and dc membrane voltage spikes have been reported [2,8]. However, novel methods were required to investigate these matters. In the 1970s, the patch-clamp (where a metal probe is inserted inside a glass tube that neutralizes ions of cell fluids with respect to external culture media) was established for single ion channel measurement. However, the glass tube opening has a diameter of 20–30 nm making it difficult to probe a single ion channel of 0.4 nm diameter. To solve this, we used a coaxial probe where the central Pt electrode has an atomic sharp needle covered by insulated glass [21]. The outer layer of a coaxial probe has a metal Au layer, ensuring that local thermal, electromagnetic and electric noises are eliminated. The most important part of this method is that it allowed us to access particular components deep inside living cells. Each biomaterial has a unique set of resonance frequencies (or band of frequencies). All known proteins and filaments resonate at particular frequencies [16,22–24]. When the coaxial probe (gold-glass-platinum) is inserted into the cell, at contact, a resonance frequency band is archived as a signature to recognize the component. Resonance frequency means a large transmission of electromagnetic signal when the sample is treated remotely with an ac signal of a particular frequency. Thus, the coaxial probe can access ultrafast signals appearing deep inside axons, dendrons, or neural branches, while a patch-clamp can be used to measure the classic nerve-based ionic bursts.

Two simultaneous measurements of signals from membrane (using the Au layer of the coaxial probe, KHz) and filaments (Pt layer of the coaxial probe, MHz and GHz) resolve transmitting signals at two distinct time domains, allowing us to address problems in neuroscience. Note that both Au and Pt layers are phase locked. Ions transmitting across membranes resonate in the milliseconds domain, and filamentary dipoles resonate at microseconds. They feed energy to each other simultaneously. The infrared spectrum in the 900 nm-1050 nm region shows that filaments deep inside a neuron acquire pulsed signals of suitable THz frequencies from thermal noise, wherefrom, filter out the microsecond bursts and transmit the singals like an antenna. Since the filaments are not metallic, absorption, emission, and reflection of electromagnetic signals occur in all directions since all three mechamisms play governing roles in energy transfer. Thus, filamentary bundles have different time domains of resonance and so do not sense neuron membranes or other components in a neural network. Membranes become transparent at the frequencies where filaments resonate and emit energy. One filamentary bundle can sense primarily other filamentary bundles of similar geometry leading to resonant energy transfer. The role of synaptic junctions is consequently insignificant. This new filamentary circuit cannot be observed using an optical microscope; the entire neural network in the culture plate is established as a single circuit of protein nanowire bundles [24]. For observation of such phenomena a dielectric microscope, where filaments resonating across the network can be imaged remotely, is required. For different time domains, different circuits are activated so that filamentary bundles vibrating on a similar time domain are part of an individual circuit. Therefore, any neuron assembly simultaneously possesses several distinct individually accessible circuits, i.e., one circuit might be studied with others considered hidden. The hidden circuits of filaments operating in the microsecond time domain have a significantly different geometry from the membrane-made neural circuits visible under an optical microscope.

Since infrared scans (5–6THz) show that these filaments are noise-driven, they do not need additional vibrational signals. Therefore, we do not need to search for electromagnetic signal generators in living cells. The triplet of triplet resonance chain that connects the smallest structures in a living body to the largest uses the shortest to the longest time domains. Therefore, any triplet band can activate the entire resonance chain [9]. As noted above, filamentary bundles create a superposition of many integrated circuits that remain silent using energy from noise. During signal transmission through a neural network, once the resonance chain activates, different frequency bands activate different filamentary circuits. It would be very interesting to observe how these circuits govern the plasticity of a neural network as a brain learns. Considering the work of E. Katz in the 1940s, ions might have been blocked but electromagnetic signals might not, so that neurons could communicate and fired. If filaments of distant, isolated neurons are connected, electromagnetic signals at a set of frequencies could arrive at filaments from distance and cause it to resonate, building and storing electrical potential [12]. Sustaining the electric potential of a nerve spike is important. A component beneath the neuronal membrane at the filamentary core holds potential bursts 10^3^ times faster than the ionic potential for buildup at the membrane. These memorized potentials could homogeneously activate the membrane at all points.

For this reason, it has been repeatedly observed that filaments fire 250 microseconds earlier than ionic nerve impulses [8,25]. Therefore, at least four filamentary bursts are made to regulate the time width of an ionic burst [25]. If two filamentary bursts are used to set the upper- and lower-time limits of an ionic burst, two other filamentary bursts ensure peak formation of the ionic burst. Thus, one of the primitive concerns regarding time gap regulation for a stream of nerve spikes is resolved. The second and most important part is shaping the circular nerve spike.

Another recent observation of neuron structure has revealed that there are 200 nm × 200 nm grids composed of actin filaments and beta spectrin just beneath the membrane of neurons, separating the membrane from the filamentary core [26]. Using a dielectric resonance microscope, it is a simple matter to image selectively the filamentary part live as an alternative to STORM [8]. Simultaneously, we have measured signal transmission through the grid layer, finding that it integrates the microseconds clocks or bursts [23,24]. It was reported that a pair of circular rings made of proteins separated by a 200 nm gap across the neural branches activated one after the other. The pair of rings were imaged live in a dielectric resonance microscope. The ring of an electromagnetic field is a vortex, and 8–10 nm below the membrane, the pair of rings can activate all the ion channels located between rings holding a homogeneous potential distribution in the entire region.

In this final section of our unique investigations of neurons, monochromatic polarized lasers were made incident on a neuron, and the reflected, refracted, and transmitted light was imaged. The nearly transparent-ordered structures inside the neuron acted as templates for light-matter interactions that imparted angular momentum to the refracted and transmitted photons. Since several ordered structures exist inside a neuron, each structure generates a distinct angular momentum. When a photon acquires an angular momentum, it projects like a ring of light and helical assemblies can affect a photon’s angular momentum in various ways. The holographic projection of each component located deep inside the neuron can be deconvoluted from the whole neuron hologram and rings of light originating from particular components can be imaged. Using an external antenna, when remote resonance frequency signals were made incident at the neuron cell, the selected component should brighten the light ring [24]. Each ring of light represents the loop-shaped electric field flow-path on the biomaterial surface; in other words, these loops are clocks. Thus, by irradiating with a laser, the 3D clock assembly of a neuron or time crystal of a neuron [5,15] is converted into a 3D assembly of light rings or optical hologram. The 3D arrangement of light rings and the 3D assembly of electromagnetic clocks are similar; therefore, the hologram shows us in real time, the dynamic variations occurring within a neuron.

Figure 2 outlines three new technologies, coaxial atom probe, dielectric resonance microscopy, and remote microwave input-optical vortex output used to find a temporal link of events occurring on different timescales from seconds to picoseconds. In these time domains, many outlandish claims have been made over the last 100 years. A plethora of data has been produced measuring clocks or rhythmic changes in biological responses. Microseconds or megahertz frequency domains had not been studied prior to our investigations, and was essentially a missing frequency band. When we assembled the spontaneously formed 3D map of clocks in all time domains, we found that different time domains act as a part of a whole pattern. Therefore, several of those works on neurons reported since the 1930s and other discarded results might be integrated into a neuron’s time crystal or the 3D clock assembly model, and challenging the prevalence of the Hodgkin Huxley paradigm.

**Figure 2.**
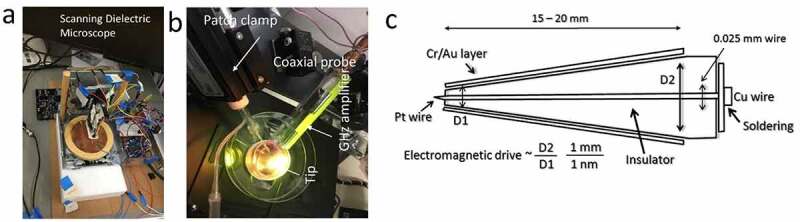
(a) Dielectric resonance microscope: biomaterials are kept on a metal or conducting plate. The tip scans through the surface, sending electromagnetic signals of various frequencies to the material and receiving the returned signal post-metal sheet reflection. (b) Coaxial probe and patch clamps are used together while measuring the filamentary firing and membrane firing. (c) The design of a coaxial probe.

## Supporting online materials

### Movie 1

With voiceover, we have shown using graphics the three major discoveries involving neurons reported in the last century but which are yet to become widely accepted. The three discoveries are integrated in a single presentation. First, Hirokawa N’s work that microtubules are densely packed inside a neuron using cryofreezing [11]; second, Xu et al.’s discovery of a 200 nm × 200 nm crystalline grid beneath the membrane [26]; finally, dielectric resonance microscopy and coaxial probe-based simultaneous measurements of membrane and filamentary communication by [1,6,8,12,13,20,22].

## Supplementary Material

Supplemental Material

## Brain rhythm database

All theoretical and experimental brain rhythm measurements from NIMS, Japan and UB, USA are available free of charge in an online database www.brainrhythm.org
